# The contribution of thymic tolerance to central nervous system autoimmunity

**DOI:** 10.1007/s00281-020-00822-z

**Published:** 2020-10-27

**Authors:** Piero Alberti, Adam E Handel

**Affiliations:** 1 Wadham College, University of Oxford, Oxford, OX1 3PN UK; 2 Nuffield Department of Clinical Neurosciences, University of Oxford, Oxford, OX3 9DU UK

## Abstract

Autoimmune diseases of the central nervous system (CNS) are associated with high levels of morbidity and economic cost. Research efforts have previously focused on the contribution of the peripheral adaptive and innate immune systems to CNS autoimmunity. However, a failure of thymic negative selection is a necessary step in CNS-reactive T cells escaping into the periphery. Even with defective thymic or peripheral tolerance, the development of CNS inflammation is rare. The reasons underlying this are currently poorly understood. In this review, we examine evidence implicating thymic selection in the pathogenesis of CNS autoimmunity. Animal models suggest that thymic negative selection is an important factor in determining susceptibility to and severity of CNS inflammation. There are indirect clinical data that suggest thymic function is also important in human CNS autoimmune diseases. Specifically, the association between thymoma and paraneoplastic encephalitis and changes in T cell receptor excision circles in multiple sclerosis implicate thymic tolerance in these diseases. We identify potential associations between CNS autoimmunity susceptibility factors and thymic tolerance. The therapeutic manipulation of thymopoiesis has the potential to open up new treatment modalities, but a better understanding of thymic tolerance in CNS autoimmunity is required before this can be realised.

## Introduction

Autoimmune disorders that affect the central nervous system (CNS) are an important cause of neurological morbidity and mortality and are associated with major economic cost [1]. The most prevalent and extensively studied of CNS autoimmune diseases is multiple sclerosis (MS), which affects ~ 2.3 million people globally with prevalence of ~ 1 in 1000 individuals in Western countries [2]. The total economic burden of MS was estimated as €14.6 billion in Europe [3]. Other CNS autoimmune conditions are divided into CNS-specific inflammatory disorders (Table 1) or systemic inflammatory disorders with CNS manifestations due to direct reaction against CNS parenchyma or CNS vasculitis.

**Table 1:** CNS-specific autoimmune diseases

Condition	Main antigens	Cellular pathogenesis	Clinical manifestations
Multiple sclerosis (MS)	Multiple possible antigens, likely myelin components	Combined B cell–mediated and T cell–mediated inflammation with innate immunity contribution	Multifocal CNS relapsing–remitting inflammatory disease; progressive neurological deficits associated with inflammation in progressive disease MS
Neuromyelitis optica spectrum disorders (NMOSD)	Aquaporin 4, myelin oligodendrocyte glycoprotein	Autoantibody-mediated	Mono-/polyphasic inflammatory disease, mainly restricted to spinal cord or optic nerves
Autoimmune encephalitis	Multiple (NMDAR, LGI1, CASPR2, AMPAR, GABA_A/B_R and others)	Autoantibody-mediated	Variable; typically involves subacute encephalopathy and seizures
Stiff person spectrum disorder (SPSD)	GAD, glycine receptor	Combined B cell and T cell involvement +/− anti-GAD antibodies	Spasms plus muscle rigidity
Rasmussen encephalitis	Unknown	T cell inflammation plus innate immunity	Progressive hemiplegia, pharmacoresistant focal epilepsy with cognitive decline
Cerebellitis	GAD, CASPR2, Yo	Variable (B and T cell)	Subacute onset of ataxia plus other clinical features
Bickerstaff encephalitis	Gangliosides (GQ1b)	Autoantibody-mediated	Brainstem deficits
CLIPPERS	Unknown	Likely T cell	Brainstem deficits responsive to steroids
Combined central and peripheral demyelination	Neurofascin	Autoantibody-mediated	Focal CNS neurological deficits plus polyradiculoneuropathy

NMDAR, *N*-methyl-d-aspartate receptor; LGI1, leucine-rich glioma-Inactivated protein 1; CASPR2, contactin-associated protein-like 2; AMPAR, α-amino-3-hydroxy-5-methyl-4-isoxazolepropionic acid receptor; GAD, glutamic acid decarboxylase; GlyR, glycine receptor; GABA_A/B_R, γ-aminobutyric acid (A or B) receptor; CLIPPERS, chronic lymphocytic inflammation with pontine perivascular enhancement responsive to steroids

**Fig. 1:**
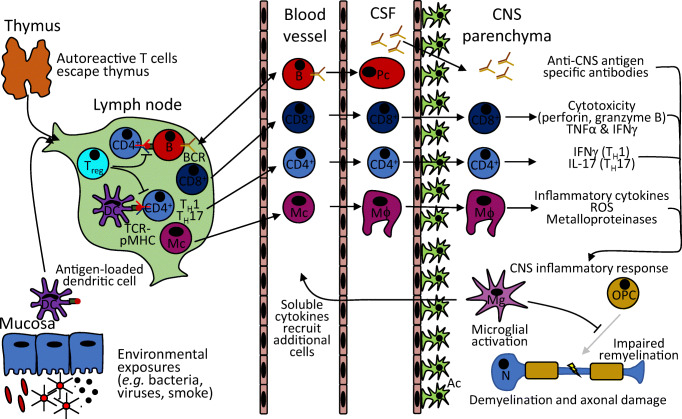
Key cellular processes leading to CNS inflammation. This is a simplified schematic of adaptive and innate immune system processes culminating in CNS inflammation. For simplicity, this outline omits important roles of many cell types, such as NKT, MAIT and γδ T cells, for which there is currently less evidence for a role of thymic selection in relation to CNS autoimmune diseases. DC, dendritic cell; Mc, monocyte; Mϕ, macrophage; Pc, plasma cell; Mg, microglia; OPC, oligodendrocyte precursor cell; N, neuron; Ac, astrocyte; TCR-pMHC, T cell receptor–peptide–major histocompatibility complex

CNS inflammation is the result of pathological dysfunction in immune tolerance, which in turn implies failure in two mechanisms which ensure that adaptive immunity recognises and responds to pathogen-associated non-self-antigens while remaining tolerant of autoantigens. Immunopathology of CNS autoimmune disorders involves breaking of tolerance in both the T and B cell compartments: CNS-directed autoreactive B cells, CD8^+^ T cells and CD4^+^ helper T cells (T_H_1, T_H_17) infiltrate the CNS along with innate immune cells leading to neurotoxicity and/or inflammatory tissue injury [4] (Fig. 1).

Tolerance in the T cell compartment is maintained by its continuous induction both centrally in the thymus and peripherally in target tissues [5, 6]. These mechanisms shape the repertoire of antigens recognised by T cells via their T cell receptors (TCR). Therefore, the nature and severity of defects in central and peripheral tolerance mechanisms determine the extent and diversity of the spectrum of autoantigens characterising different CNS autoimmune diseases [7]. B cell tolerance is established by sequential checkpoints in both early and late stages of B cell differentiation in bone marrow [8, 9]. While this central B cell tolerance develops independently of T cell modulation, autoreactive B cells escaping negative selection in bone marrow or generated in the periphery as a result of somatic hypermutation are normally prevented from causing autoimmunity via B-T cell interaction, most importantly induction of B cell anergy or FoxP3^+^ regulatory T cells (T_reg_) [10, 11].

Experimental and clinical studies on the role of immune tolerance in the onset and progression of CNS autoimmunity have predominantly focused on mechanisms underpinning establishment and long-term maintenance of *peripheral* tolerance [12]. Historically, the contribution of thymic tolerance mechanisms in both the emergence and continuance of CNS inflammation has not been as extensively studied. However, mounting in vitro and in vivo evidence has reignited interest in mechanisms of central tolerance, particularly thymic selection, in the pathogenesis of CNS autoimmune diseases [13].

In this article, we review current models of the molecular and cellular mechanisms of thymic central tolerance and their role in CNS autoimmunity, review current preclinical and clinical evidence for involvement of thymic dysfunction in CNS autoimmunity and finally consider the potential for therapeutic monitoring and targeting of central tolerance as an avenue to develop novel treatments for patients suffering from MS and other autoimmune CNS diseases.

## Thymic development and function

The thymus develops as part of the segmentation of the posterior pharynx: all TECs originate from the ventral endodermal lining of the third pharyngeal pouch. This primordial thymic anlage attracts early thymocytes and develops into distinct cortical and medullary regions where the interaction of TECs with other local antigen presenting cells (APCs) and stromal cells forms a complex 3D scaffold crucial to thymocyte differentiation and selection [14].

**Fig. 2:**
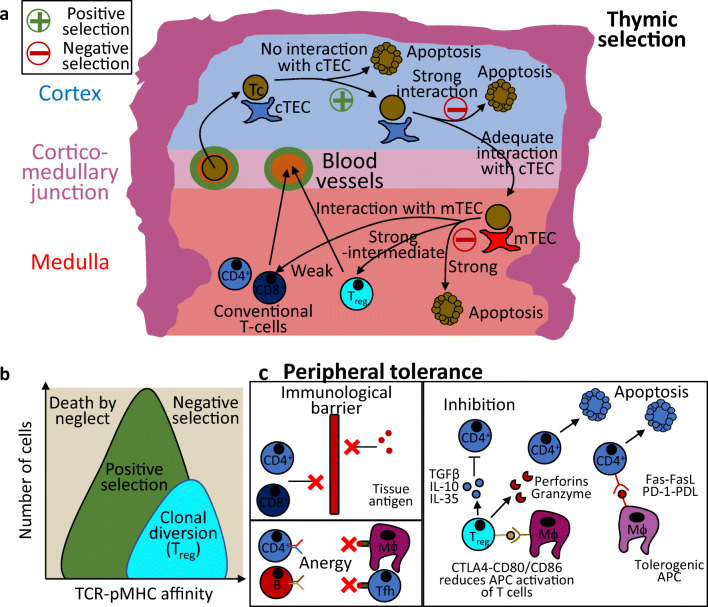
Thymic and peripheral tolerance mechanisms. **a** An overview of thymocyte (Tc) and thymic epithelial cell (TEC) interactions within the thymus. **b** The thresholds of affinity model of thymic selection of thymocytes. **c** An overview of peripheral tolerogenic mechanisms

Differentiation, functional specialisation and establishment of tolerance of developing T cells (thymocytes) depend on their interaction with thymic epithelial cells (TECs) (Fig. 2a). TECs are MHC-expressing antigen-presenting cells (APCs) whose interaction with thymocytes restricts the T cell repertoire to conventional αβT cells expressing TCRs which functionally engage self-MHC (positive selection) without leading to autoreactivity (negative selection) [15]. Additionally, growth factor and cytokine signalling by TECs supports thymopoiesis and influences thymocyte lineage specification [16, 17].

As well as conventional αβT cells, the thymus also produces γδT cells, natural killer T (NKT) cells and mucosal-associated invariant T cells. These are not discussed in depth here, but all are associated with CNS autoimmune disease and require intact thymopoiesis for their development [18–20].

Positive selection of conventional T cells occurs in the cortex and is mediated exclusively by cortical TECs (cTECs). Thymocytes are selected by stromal survival signals if they express a TCR with high affinity for its cognate peptide-MHC (pMHC) complex expressed on cTEC surfaces. Thymocytes that do not fulfil these criteria of MHC restriction (around 98%) are prohibited from further maturation into T cells by withdrawal of selective stromal survival signals and die by neglect.

Negative selection of thymocytes occurs in both the cortex and medulla [21, 22]. It is mediated by cTECs or mTECs together with other intrathymic APCs. In negative selection, antigen presentation induces apoptosis of thymocytes expressing TCRs with high affinity for autoantigens [23]. At the same time, thymocytes expressing TCRs with intermediate to high affinity for autoantigens, undergo a process of clonal diversion: engagement of autoantigens by their self-reactive TCR in presence of a favourable cytokine milieu (TGFβ, IL-2) induces FoxP3 expression and differentiation into thymic T_reg_ (tT_reg_), which limit peripheral T cell autoreactivity [24–27].

The balance between survival, clonal diversion and clonal deletion is principally determined by the affinity of TCR-pMHC interactions.

### Molecular mechanisms of negative selection

Establishment of thymic central tolerance by negative selection is dependent on the ability of thymocytes to respond differentially to the specific kinetics of TCR-pMHC binding. For example, TCR-pMHC complex binding triggers Ca^2+^ influx and extracellular-signal regulated kinase (ERK) activation: prolonged, low-level Ca^2+^ influx and ERK signalling maintains thymocyte survival, whereas rapid and robust ERK activation triggers clonal deletion [28]. Moreover, close to thymic selection thresholds (Fig. 2b), small quantitative increases in TCR ligand affinity and binding time trigger a qualitative shift in the phosphorylation status and subcellular compartmentalisation of Ras/MAPK signalling intermediates, whose recruitment to thymocyte membrane signals induction of negative selection [29].

Thymic negative selection requires that TECs present peptides derived from virtually all genes expressed within the body, a process known as promiscuous gene expression (PGE). mTECs use molecular pathways coordinated by the proteins AIRE (~ 4000 genes) and FEZF2 (~ 400 genes) to drive the expression of tissue-restricted antigens (TRA) and ensure that positively selected thymocytes are screened against a wide complement of self-peptides [30–32]. Genes regulated by AIRE-mediated PGE are associated with high levels of chromatin marks that characterise transcriptional repression.

The molecular orchestration of PGE in TEC is essential for the negative selection of thymocytes. However, TECs alone are insufficient to induce complete negative selection [33].

### TEC-independent negative selection

Thymic APCs other than TECs, mainly intrathymic B cells and dendritic cells (DCs), also play an active role in TRA presentation. Firstly, intrathymic B cells directly participate in PGE. Circulating naïve B cells that immigrate into the thymus interact with cognate autoreactive CD4^+^ thymocytes via CD40: this leads to MHC-II and CD80 upregulation and “licences” B cells for AIRE expression, allowing TRA presentation and central tolerance induction [34]. Thymic B cells also contribute to clonal diversion into tTreg, and mTEC function and TRA presentation by lymphotoxin secretion [35, 36]. DCs participate in negative thymocyte selection by three main mechanisms: thymic DCs present autoantigens found in thymic parenchyma or the medullary perivascular system [37]; circulating active DCs are recruited to the medulla to present autoantigens from peripheral tissues [38, 39]; thymic DCs present TEC-derived autoantigen through exosomal transfer [40]. DCs are known to present some encephalitogenic T cell epitopes to thymocytes, which may have implications for CNS autoimmunity [41, 42].

### Failure of negative selection

A failure of negative selection is a requirement for peripheral T cell autoreactivity in both CNS autoimmunity and other organ-specific autoimmune diseases. Even under physiological conditions, TRA presentation to thymocytes is imperfect and permits potentially autoreactive T cells to escape to the periphery [43, 44].

In most circumstances, peripheral tolerance is able to compensate for incomplete thymic negative selection. This occurs through three main processes: intrinsic or acquired immune privilege [45], a key determinant of the relative contribution of thymic dysfunction to CNS autoimmunity; induction of T cell anergy [46]; and suppression of T cell responses by T_reg_ (Fig. 2c) [11, 47].

However, peripheral tolerogenic mechanisms cannot completely compensate for defective negative thymic selection, as illustrated by autoimmune polyendocrine syndrome type 1.

### Autoimmune polyendocrine syndrome type 1 (APS-1)

Congenital loss-of-function AIRE mutations lead to the severe dysimmune manifestations observed in autoimmune polyendocrine syndrome type 1 (APS-1): hypoparathyroidism, adrenal insufficiency and chronic mucocutaneous candidiasis [48–50]. Many of these clinical manifestations are attributable to defective negative selection of thymocytes by mTEC. However, AIRE is also detectable in other cell types both within the thymus (AIRE-expressing B cells) and elsewhere (AIRE-expressing dendritic cells) [34, 51]. Evidence for the role of these non-mTEC AIRE-expressing cells in tolerance is controversial, particularly since recent data on the expression profile of human extrathymic AIRE-expressing dendritic cells suggest that AIRE does not drive TRA expression [52, 53].

Despite the aberrant T cell selection observed in APS-1, it is rare for autoimmune manifestations to affect the CNS beyond the pituitary gland [54, 55].

### CNS antigen presentation in the thymus

**Fig. 3:**
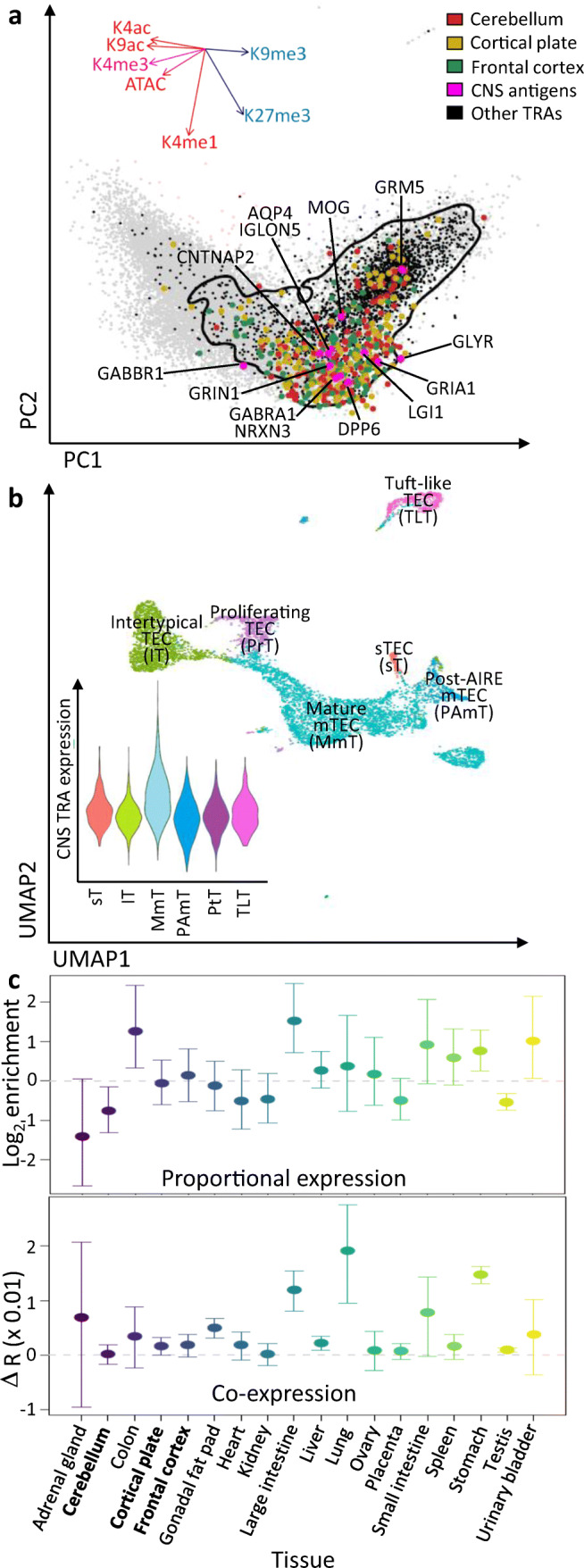
Molecular characteristics of CNS antigen expression in the thymus. **a** A principal component plot of chromatin modifications in mature mTEC, highlighting CNS TRAs from cerebellum, cortical plate and frontal cortex. The positions of gene orthologues for known antigenic targets in CNS autoimmunity are labelled. The inset shows the contribution of each chromatin modification to the first and second principal components. The contour shows the 95% confidence interval for the kernel density of non-CNS TRAs. **b** A Uniform Manifold Approximation and Projection plot of single-cell RNA-seq TEC data (gated to exclude cTEC). The inset shows the overall module expression levels for CNS TRAs in each TEC subpopulation. TEC annotation was performed by transferring labels from an annotated dataset of TEC using Seurat [58, 59]. **c** Plots of log_2_ enrichment for proportional expression and differential co-expression of TRAs within mature and post-AIRE mTECs relative to permuting TRA organ specificity labels 1000 times. Error bars show empirical 95% confidence intervals. TRA organ specificity was defined as in [56]. Data was derived from [56, 57]

The presence of peripheral, CNS-reactive T cells is difficult to reconcile with the lack of neuronal autoimmunity in APS-1. One possibility is that particular aspects of how CNS-specific TRAs are presented to thymocytes by mTEC and other thymic stromal cells may explain the relative lack of CNS manifestations in APS-1. TRAs in general have characteristic patterns of chromatin modifications, depleted for active chromatin (e.g*.* H3K4me3) and enriched for repressive chromatin (e.g. H3K27me3). CNS TRAs showed very similar chromatin modifications to other TRAs (Fig. 3a). Overall, CNS TRAs showed the highest expression within the population of mature mTEC (Fig. 3b). There was no evidence that the proportional expression or co-expression patterns of CNS TRAs were significantly different from TRAs of other tissues, suggesting that excessive redundancy in the expression of CNS antigens within the thymus is unlikely to account for the low frequency of CNS manifestations in APS-1 [13, 56–58] (Fig. 3c). It is possible that a mismatch between transcript expression and peptide abundance may explain part of this discrepancy; future studies are likely to examine this directly.

One characteristic that distinguishes CNS-specific TRAs from those found in other organs, is an enrichment for micro-exons: the expression of exonic sequences ≤ 30 base-pairs in length [60, 61]. Relatively little is understood regarding the expression of micro-exon-containing genes within mTEC, but recent research suggests that micro-exons are poorly represented within the thymus with *or* without the expression of AIRE [62]. CNS antigens are also known to undergo post-translational modification [63]. In a similar fashion to micro-exons, the representation of post-translationally modified TRA in thymus is incomplete [64]. The implications of these unique antigenic characteristics for CNS autoimmunity are currently unclear but would constitute potential routes by which developing thymocytes reactive against CNS TRAs could escape negative selection.

Overall, there are no characteristics of the thymic expression pattern of CNS-specific antigens that would explain why the CNS compartment is rarely affected in APS-1 patients.

### CNS-autoreactive T cells

T cells contribute to the immunopathogenic mechanisms of CNS autoimmune diseases both via cellular immunity, involving direct induction of CNS inflammation along with innate immune cells, and humoral immunity, stimulating autoantibody production in B cells [65]. Evidence that T cells can result in CNS immune-mediated damage is provided by either CNS-reactive T cells in patient CNS tissue and/or cerebrospinal fluid (CSF) samples or association between HLA haplotypes and susceptibility to CNS autoimmune disease.

Neuropathological studies show autoreactive T cell infiltration is a feature of both paraneoplastic forms of autoimmune encephalitis, in which cytotoxic T cells and “onconeural” Abs are directed against intracellular CNS autoantigens, and autoimmune encephalitides characterised by autoantibodies against CNS surface self-antigens, e.g. NMDA receptors, leucine-rich glioma-inactivated protein 1 (LGI1) [66–69]. Similarly, in stiff person syndrome spectrum disorders (SPSDs), IFNγ-producing CD4^+^ T cells specific for an isoform of autoantigen glutamic acid decarboxylase (GAD65) can be isolated from patient blood and CSF samples and, in ~ 90% of patients, stimulate production of anti-GAD65 IgG autoantibodies by intrathecal B cells [70, 71]. In Rasmussen encephalitis, there is demonstrable infiltration of CNS parenchyma by IFNγ-producing CD8^+^, CD4^+^ and γδ-T cells, although as of yet without an identifiable autoantigenic target [72, 73].

Beyond a direct role of T cells in autoimmune-mediated CNS toxicity, T cell help in the form of cytokines (e.g. IL-2) and co-stimulatory molecules (e.g. CD40 ligand) has been identified as an important stimulant of B cell autoantibody production in patients with neuromyelitis optica spectrum disorders (NMOSD) and NMDA-receptor antibody encephalitis [74, 75].

In anti-PIT-1 antibody syndrome, a thymoma-associated form of autoimmune hypophysitis characterised by acquired GH, TSH and prolactin deficiency, both direct T cell–mediated neurotoxicity and T cell humoral responses have been identified. There was CD8^+^ T cell infiltration of pituitary and other endocrine organs [76]. The correlation between levels of circulating anti-PIT 1 antibody and aberrant PIT-1 expression in thymomas supports the role of dysfunctional thymic selection in anti-PIT-1 antibody syndrome [77].

Association of HLA haplotypes with increased risk of CNS autoimmune disease also implicates T cell effects as key drivers of CNS inflammation. There is a > 90% association of the HLA-DRB1*07:01 allele with susceptibility to LGI1 antibody encephalitis, as well as a ~ 50% association of the HLA-DRB1*11:01 allele with susceptibility to contactin-associated protein-like 2 (CASPR2)–mediated CNS autoimmune diseases [78]. This association with MHC class II HLA alleles demonstrates that autoantigen presentation to T cells is a key process in the pathogenesis of LGI1 and CASPR2 antibody encephalitis.

Overall, these findings strongly indicate thymic escape of autoreactive T cells as an important pathophysiological mechanism in autoantibody-mediated CNS autoimmune diseases. However, the rarity of CNS manifestations in APS-1 argues that a failure of thymic selection alone is unlikely to be sufficient for CNS autoimmunity.

## Evidence for thymic tolerance in CNS inflammation

The most commonly used model of CNS autoimmunity is experimental autoimmune encephalomyelitis (EAE): induction of cerebral and spinal inflammation by myelin autoantigens, such as myelin oligodendrocyte glycoprotein (MOG), proteolipid protein (PLP) and myelin basic protein (MBP). This system has been widely used to model the key features of human MS, albeit with several key clinical and pathophysiological differences between human CNS autoimmunity and EAE [79, 80].

### AIRE-dependent tolerance in CNS autoimmune disease

Induction of EAE in AIRE-deficient mice can provide insight into the potential role of thymic tolerance in CNS autoimmunity. The susceptibility of *Aire*^*−/−*^ mice to EAE is age-dependent and correlates with the gradual reduction of an initially elevated number of T_reg_ cells in the brain parenchyma [81, 82]. AIRE deficiency results in altered development and thymic recirculation of T_reg_ as well as the inappropriate diversion of T_reg_ into an effector T cell phenotype [83–85]. However, further studies in MHC humanised, *Aire*^−/−^ mice showed that, even with peripheral T_reg_ depletion, spontaneous CNS inflammation did not develop [86].

Overall, this supports clinical insights from APS-1 patients suggesting that impairment of thymic selection alone is insufficient for the development of CNS autoimmunity.

### T_reg_ cells in CNS autoimmune disease

Thymic T_reg_ (tT_reg_—those T_reg_ generated in the thymus) and peripheral T_reg_ (pT_reg_—peripheral conversion of effector T cells into T_reg_) cells have distinct roles in control of CNS inflammation [12]. Several studies show that T_reg_ cell–mediated immunomodulation has a pivotal role in protection from EAE and MS by suppression of peripheral myelin-reactive, potentially encephalitogenic T cells [87]. Acute depletion or functional inhibition of circulating T_reg_ cells in animal models exacerbates EAE course, and clinical studies suggest that T_reg_ cells in MS patients display defects in effector T cell suppression [88–90]. Presentation of neuronal antigenic material by DCs to autoreactive T cells in a non-inflammatory context leads to their differentiation into Hopx^+^ pT_reg_ cells, which in turn provide long-lasting tolerance that protects from subsequent EAE induction [91]. MS patients exhibit defects in peripheral B cell tolerance in spite of normal central B cell selection, implicating dysfunction in T_reg_ cell–mediated modulation of peripheral B cell differentiation checkpoints [92]. Recent results from murine models have also identified a role of T_reg_ cells in promoting oligodendrocyte progenitor cell proliferation and remyelination [93].

tT_reg_ cells appear to be primarily involved in recovery from CNS autoimmunity. Spontaneous resolution of EAE in mice has been shown to involve accelerated tT_reg_ cell proliferation, differentiation and thymic output and is effectively prevented by thymectomy [94].

Systemic loss of T_reg_ cell function due to mutations in the T_reg_ master regulator, *FOXP3*, leads to immune dysregulation, polyendocrinopathy, enteropathy, and X-linked (IPEX) syndrome, characterised by severe multi-organ autoimmunity [95, 96]. However, as with defective thymic selection in APS-1, CNS involvement in patients with IPEX syndrome is rare, with only one report of posterior reversible encephalopathy syndrome (PRES) [97].

Overall, this suggests that importance of T_reg_ cells in CNS autoimmunity reflects a combination of regenerative and immunomodulatory functions but that the loss of T_reg_ peripheral tolerogenic functions is insufficient to induce spontaneous CNS autoimmunity.

### Insight from genetic studies

Several lines of genetic evidence highlight the necessity of normal thymus function to maintain homeostasis between effector and tolerogenic mechanisms of adaptive immunity in peripheral tissues, including the CNS. Mutations in genes required for thymus development and/or thymocyte selection have been associated with CNS autoimmunity or vice versa.

Development, differentiation and function of TECs require transcriptional master regulator *Foxn1*. *MAP3K14* and *IRF8*, genes whose mouse orthologues are regulatory targets of *Foxn1*, have been implicated in MS by genome-wide association studies (GWAS) [98–100]. Conditional *Foxn1* ablation in mice, which accelerates thymic involution, reduces *Aire* expression and disrupts negative selection, also induces autoimmune infiltration of pro-inflammatory cells in the CNS [101]. TEC-specific knockout of *Map3k14* in mice leads to drastic decrease in thymic development and IL-17 secretion of dendritic epidermal γδ T cells, as a result of downstream loss of expression of *Rorc* and *Il23r*, genes required for IL-17 synthesis in γδ T cells [102]. As clinical studies have reported increased frequency of IL-17-producing γδ T cells in the CSF of MS patients, abnormal γδ T cell development may be important to the pathogenesis of CNS autoimmune disease [18]. Finally, *Irf8* is part of a transcriptional program that facilitates *Aire* expression in TECs; thus, *Irf8* dysfunction may alter representation of AIRE-regulated CNS autoantigens in the thymus [103, 104].

Genetic evidence also points to associations between dysfunction in mechanisms of thymic selection and CNS autoimmunity. *CLEC16A*, variants of which are associated with susceptibility to MS, is involved in the control of TEC autophagy, a process that regulates MHC-associated thymic presentation of lysosomal, nuclear and mitochondrial peptide antigens [105, 106]. Silencing of *Clec16a* protects against autoimmunity by inducing CD4^+^ T cell hyporeactivity [106], and *CLEC16A* expression is upregulated in peripheral APCs of MS patients [107]. EAE severity in mice is exacerbated by dysfunction in *PRSS16*, which encodes a serine protease controlling peptide presentation to developing thymocytes [108, 109].

GWAS over the last decade have identified over 200 gene loci that independently contribute to MS pathogenesis [110]. Like many putative autoimmune diseases, major genetic variants associated with MS occur in the MHC class II subgroup of the HLA complex: HLA-DRB1*15:01-containing haplotypes carry the strongest association with MS risk [111]. Yet, the extent to which these variants affect thymic tolerance is difficult to determine, as specific haplotypes will both exert intrathymic effects on thymocyte selection and influence peripheral mechanisms of antigen presentation [112]. Autoimmunity-associated MHC polymorphisms are typically thought to alter TCR-pMHC complex binding dynamics and may cause extensive TCR-pMHC microcluster formation leading to escape of autoreactive T cells [113, 114].

In general, characterisation of genetic pathways associated with thymic function is less comprehensive than pathways associated with peripheral innate or adaptive immune cells. Variants in other genes associated with susceptibility to MS or other CNS autoimmune diseases might hence affect central thymic tolerance processes in addition to their presently understood roles in peripheral immunity [110]. Emerging sequencing datasets from thymic stromal cells derived from murine or human samples will help annotate biological pathways important for thymic function [115, 116].

## Evidence from animal models

### Responses to myelin autoantigens in mouse models of CNS autoimmunity

The pathological effects of manipulating thymic expression of CNS antigens in murine EAE provide insight into the roles of thymic selection in CNS inflammation. Immunisation with PLP in *Plp1*^*ΔTEC*^ mice, which lack thymic expression of resistance-associated PLP isoforms, leads to a more severe EAE course than in *Plp1*^*WT*^ mice [41]. This is consistent with previous studies showing that TEC expression of an encephalitogenic PLP splice isoform induces T cell tolerance for all PLP epitopes in EAE-resistant but not EAE-susceptible inbred mouse strains [117].

Similar protective effects of *Mbp* and *Aqp* thymic expression are seen in EAE models induced by MBP and AQP4 respectively, with peripheral expansion of CNS-reactive T cells not seen in wild-type mice [118, 119]. Bypassing thymic tolerance via neonatal adoptive transfer into EAE-resistant rats of resting MBP-reactive T cells induces susceptibility to later EAE induction by MBP, despite MBP expression in TECs [120]. Similarly, transfer of T_H_17-polarised AQP4-specific cells into wild-type mice induces clinical and histologic signs of CNS autoimmune disease consistent with NMOSD [119].

Collectively, evidence suggests that experimental models of defective thymic selection can overwhelm peripheral tolerance mechanisms so that CNS-reactive T cells generate CNS autoimmunity. However, since these animal models do not develop spontaneous CNS inflammation in the absence of exogenous antigenic priming, it is unlikely that these models could completely recapitulate the complex pathophysiology of MS.

### Thymic dysfunction and the rarity of CNS inflammation

This evidence of apparently dominant influences of central tolerance in some EAE models further highlights the paradox in the lack of spontaneous CNS autoimmunity in human (APS-1) and animal (*Aire*^*−/−*^ mice) models in which thymic selection is entirely absent. Two hypotheses have been advanced to explain this apparent contradiction.

The traditional view that the blood-brain barrier (BBB) entirely prevents infiltration of CNS-reactive T cells, making the CNS a perfectly immunoprivileged site, has been abandoned in light of current evidence [121]. In vivo tracing and imaging findings show that, under physiological conditions, T cells of heterogenous TCR specificities, including CNS-reactive T cells, frequently cross the BBB of meningeal and CNS parenchymal vessels to scan leptomeningeal and perivascular spaces for APC-presented antigen, suggesting that continuous *trans*-BBB immune cell trafficking is important to homeostatic immune surveillance of CNS parenchyma [122–124]. Recent findings have shown that the CNS harbours a complex network of T cells, which includes resting myelin-reactive T cells, involved in recovery from brain injury, CNS ageing and neurodegeneration, and higher cognitive function [125–128]. Disruption of homeostatic T cell surveillance is central to CNS inflammation [129]. Moreover, induction of BBB breakdown in the context of stroke rapidly induces clonal expansion of CNS-reactive T cells in brain parenchyma, indicating that partial immune privilege is important for the normal protection from CNS autoimmunity [130].

As well as this efferent arm (i.e. immune-cell CNS entry), the afferent arm of the neuroimmune axis influences roles of central and peripheral tolerance in CNS autoimmunity. The lymphatic system of the CNS, which includes glymphatic clearance of interstitial CNS solutes by CSF, drains soluble antigens and immune cells from CSF and CNS parenchyma into the cervical lymph nodes (CLNs) [131, 132]. The CNS lymphatic system constitutes a key site where CNS antigen presentation may trigger activation of CNS-reactive T cells. In rodents, antigens injected in CSF or brain parenchyma gradually accumulate in deep CLNs [133]. Both pharmacological ablation of meningeal lymphatics and dCLN resection attenuate EAE development, most likely by reducing licensing, reactivation and acquisition of encephalitogenic profile of CNS-reactive T cells in secondary lymphoid tissues [134, 135].

An additional explanation for the rarity of CNS inflammation in the context of thymic dysfunction is that antibodies against pro-inflammatory cytokines may preferentially block peripheral pathogenic processes leading to CNS autoimmunity. APS-1 patients, which lack T cell–dependent B cell tolerance, harbour high-affinity, strongly disease-ameliorating autoantibodies against type I interferons, which may account for the relative absence of CNS involvement in APS-1 [136].

Overall, it is likely that the combination of relative immune privilege and a constitutive anti-inflammatory state makes the CNS, under normal conditions, sufficiently resistant to induction of autoimmunity in spite of thymic escape of potentially encephalitogenic T cell clones. This suggests that additional exogenous triggers are required to break peripheral tolerance in human CNS autoimmunity.

### Environmental triggers of CNS autoimmunity

Animal models of CNS autoimmunity require exogenous priming with CNS antigens to break central and peripheral tolerance. Molecular mimicry between pathogen-derived antigens and autoantigens, leading to priming and later cross-activation of autoreactive T (and B) cells, may be a critical environmental factor required, in addition to thymic dysfunction, to trigger CNS autoimmunity under physiological conditions [137].

The strongest association between MS susceptibility and an environmental pathogen is with Epstein-Barr virus (EBV) [138, 139]. Crystallographic studies have revealed strong structural homology between TCR epitopes of HLA-DRB1*15:01-restricted MBP and HLA-DRB5*01:01-restricted EBV peptides, suggesting that molecular mimicry may be a key determinant of the MHC class II link in MS [140]. Moreover, EBV-specific T_H_1 cells from infected MS patients can cross-react against MBP peptides [141].

Molecular mimicry between AQP4 and *Clostridium perfringens* has been implicated as an environmental factor in NMOSD. NMO patients can harbour AQP4-specific T_H_17-polarised cells cross-reactive against *C. perfringens* antigens, and *C. perfringens* is overrepresented in their gut microbiome [142, 143].

Distinct from molecular mimicry, infections with a variety of pathogens can alter the phenotype and reactivity of cells migrating into the CNS [144, 145]. This is an epitope-independent mechanism by which environmental exposure could overwhelm peripheral tolerance in the presence of potentially autoreactive T cell clones that escaped thymic selection.

## Evidence from human autoimmune disease

Due to clear differences in the pathophysiology of EAE and MS, findings from EAE in murine models cannot be directly extrapolated to human CNS autoimmune disease [79]. Importantly, the contribution of ongoing thymopoiesis to adaptive immunity differs between rodents and humans. In mice, robust thymic function allows lifelong thymocyte selection and naïve T cell output, whereas in humans, thymic involution starts in the second year of life [146]. Therefore, in contrast to mice, in adult humans adaptive immune function is mainly maintained by peripheral homeostatic proliferation of naïve and memory cells in an established T cell compartment. The early involution of human thymic tissue and its relative inaccessibility to clinical investigation have been major obstacles to studies of links between loss of thymic tolerance and CNS autoimmunity. Nonetheless, several lines of indirect evidence from patient studies point to potentially important roles of dysfunctions in thymic tolerance in human CNS autoimmune disease.

### Recent thymic emigrant cells and TREC measurements

Quantification of T cell receptor excision circles (TRECs) is an indirect assay of thymic activity that circumvents tissue accessibility problems. TRECs are circular non-replicating DNA fragments, produced as a result of V(D)J recombination of TCR chain loci during thymocyte development, which can be detected in peripheral blood T cells [147–149]. The number of TRECs per million peripheral T cells correlates to the proportion of de novo T cells that have recently emigrated from the thymus and have not yet undergone substantial peripheral homeostatic proliferation.

**Fig. 4:**
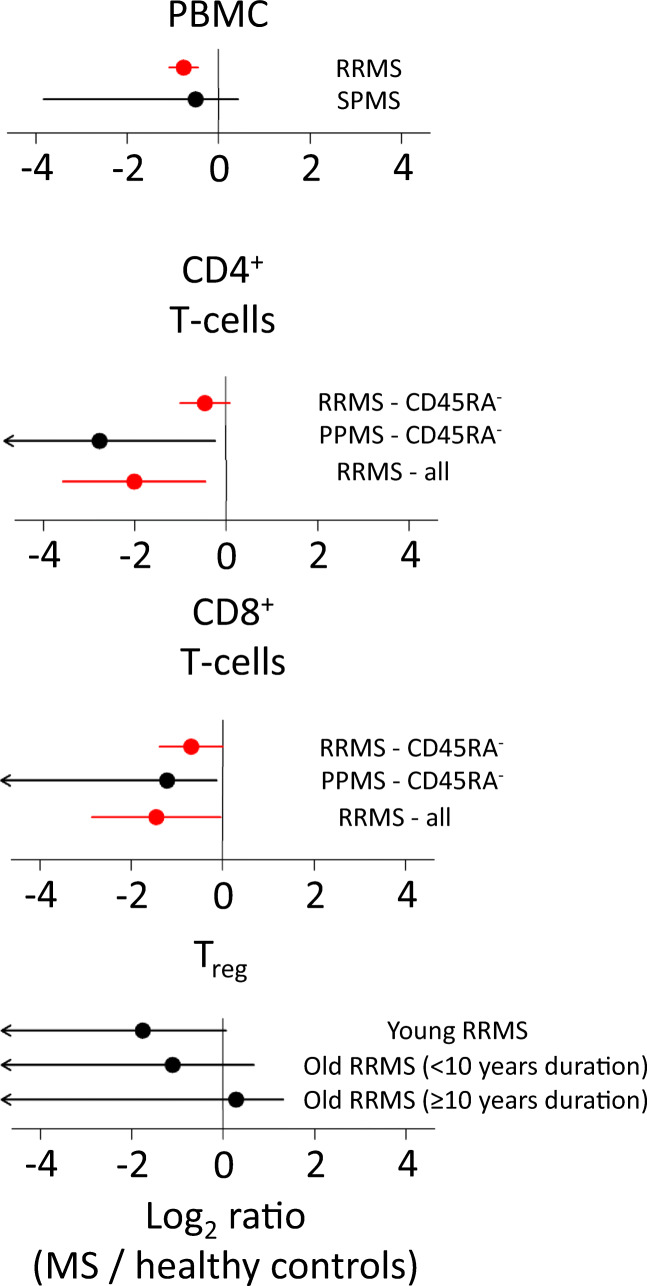
Measuring T cell receptor excision circles in MS. A schematic of selected elements of thymopoiesis. Log2 ratios of T cell receptor excision circle abundances between patients with MS and healthy controls are shown in forest plots. Red points indicate meta-analysed values inferred from a random effects model weighted by inverse variance. Standard errors were estimated from data presented in each study. RRMS, relapsing remitting MS; PPMS, primary progressive MS; SPMS, secondary progressive MS; T_reg_, regulatory T cells; PBMC, peripheral blood mononuclear cells. The scale bar indicates the magnitude of the log2 ratio

Several studies have compared numbers of TRECs in peripheral T cells of MS patients versus healthy controls to determine the influence of thymopoiesis on the peripheral T cell clonal expansion found in MS [150–161]. Meta-analysis of these findings reveals a remarkably consistent reduction of TREC numbers in different subsets of peripheral lymphocytes in MS, which can be interpreted as a sign of accelerated or premature thymic senescence (Fig. 4). However, it is important to note that low TREC numbers do not unequivocally reflect reduction in thymic output of naïve T cells, as increases in peripheral T cell proliferation will also dilute the proportion of recent thymic emigrants (RTEs) in the T cell compartment [162]. As not all TREC studies in MS patients sort T cell based on phenotypic markers, these do not provide conclusive evidence that MS-related decreases of TREC levels are due to reduced thymic output instead of homeostatic or antigen-induced T cell proliferation.

CD31 has been used as a selective marker to distinguish CD31^+^ TREC^hi^ RTE CD4^+^ T cells from CD31^−^ TREC^lo^ CD4^+^ T cells that have undergone homeostatic proliferation. The frequency of CD31^+^ RTE cells in blood are reduced in MS patients relative to healthy controls [157–161, 163]. Furthermore, paediatric MS patients exhibit significantly reduced levels of circulating CD31^+^ RTE effector T cells and T_reg_ cells relative to healthy controls as well as adult-like naïve/memory T cell ratios [164]. In addition, MS-associated genetic variants of *IL7RA*, which promotes early thymocyte survival, were associated with an increased frequency of RTEs [165]. These lines of evidence implicate premature intrinsic failure of thymopoiesis as a key factor in early onset of CNS autoimmunity.

### Clonal expansion of CNS-reactive T cells and TCR chain pairings

As discussed above, CNS-reactive T cells are a central factor in the pathophysiology of human CNS autoimmune disease. Clonally expanded populations of peripheral CNS-reactive T cells, characterised by increased proliferation and pro-inflammatory cytokine release (IFNγ, IL-17, GM-CSF) in response to CNS antigens relative to healthy controls, have been observed in patients with MS, NMOSD and neuropsychiatric systemic lupus erythematosus (SLE) [44, 142, 166].

Clonally expanded CNS-reactive T cell populations can be related to thymic selection defects by population-level high-throughput sequencing of TCR locus rearrangements, which allows to detect overrepresentations of TCR chain pairings in the peripheral T cell compartment. T cells from MS patients have more shared clonal TCRβ chain sequences between CNS, CSF and peripheral T cell pools than healthy controls [167, 168]. Longitudinal TCR sequencing has shown that clonally expanded T cell populations could be detected in brain lesions, CSF and blood samples of a single patient with MS over an 18-year course, strongly suggesting that thymic escape of CNS-reactive T cells contributes to the peripheral pool of encephalitogenic T cells with subsequent maintenance by homeostatic proliferation [169].

## Roles of MS risk factors in thymopoiesis

Indications of a link between the thymus and CNS autoimmune diseases have also come from analysis of MS risk factors. Some factors known to be associated with MS susceptibility, such as EBV infection, vitamin D levels, female sex and certain inflammatory or metabolic influences, have also been linked to alterations in thymopoiesis.

### EBV infection

EBV infection is strongly associated with susceptibility to MS [138, 139]. There is also evidence to suggest that EBV infection may strengthen links between thymic escape of autoreactive T cells and CNS pathology. Specifically, EBV infection of B cells in vitro increases secretion of chemokine CCL17 [170]. In turn, CCL17 in the thymus has been shown to influence differentiation and cytokine profiles of tT_reg_ cells [171]. Moreover, interaction between CCL17 and its receptor CCR4 promotes pathogenesis of both EAE and MS by stimulating *trans*-BBB transmigration of T_H_17 cells [172, 173]. However, the extent to which EBV infects cells within the thymus is controversial [174–177]. Collectively, data on the role of CCL17 provides tentative evidence that direct EBV infection of the thymus could alter thymic output and thus CNS inflammation.

### Vitamin D deficiency

Low serum levels of 25-(OH)-vitamin D, particularly in utero, in early life or during adolescence, are associated with increased risk of MS [178–183]. Exposure to low seasonal low levels of vitamin D either in utero or early in life has been proposed to underlie the month-of-birth effect on MS susceptibility [184, 185]. Maternal vitamin D deficiency is indeed associated with reduced foetal thymic volume [186]. Low vitamin D levels later in life are not associated with reduced thymic output but are correlated with the proportion of tT_reg_ in the periphery [187, 188]. Furthermore, thymic output, as measured by TREC levels, is correlated with month-of-birth [189], supporting a potential link between thymic output, vitamin D and MS susceptibility.

### Sex-related risk factors

As observed in many other autoimmune diseases, MS risk is clearly associated with female sex [190]. It is likely that this female sex bias in MS susceptibility may involve sex-dependent endocrine effects on thymic tolerance. Studies in rodents show that exposure to high oestrogen levels induces premature thymic atrophy associated with depletion of thymus-homing progenitors and reduced DN thymocyte proliferation, apoptosis of DP thymocytes and downregulation of AIRE expression with subsequent impairment of PGE in mTECs [191–195]. In contrast, androgen exposure upregulates thymic AIRE expression, leading to increased TRA presentation by TECs and thereby reduced EAE susceptibility via a male sex–dependent and AIRE-mediated process [196]. Sex hormone–related effects on thymic selection are therefore highly likely to contribute to the strong association between female sex and MS susceptibility.

### Inflammatory and metabolic risk factors

Inflammatory and metabolic alterations associated with cigarette smoking and high body mass index (BMI) during childhood and adolescence, both of which constitute risk factors for MS [197, 198], have also been tentatively linked to disruption in thymic function. Maternal smoking has been linked to reduced neonatal thymic size, and prenatal nicotine exposure in mice results in persistent thymic hypoplasia with a reduction in CD4^+^ SP thymocytes [199, 200]. Imaging studies also show that smoking and high BMI in adults are associated with premature fatty involution of the thymus [201, 202].

Overall, evidence that risk factors of CNS autoimmune disease are associated with thymic dysfunction is considerable but mainly indirect. Further research should thus focus on the effects of genetic risk *loci*, lifestyle factors and environmental exposures on alterations in thymic function [203].

## Evidence from alterations in thymic function

As well as evidence that CNS autoimmune disease can be associated with thymic dysfunction, several studies have shown that direct disruption of thymic tolerance due to thymoma or thymectomy can be associated with alterations in CNS autoimmunity.

Thymic tumours can be associated with development of paraneoplastic autoimmune encephalitides involving CNS infiltration of cytotoxic T cells and onconeural autoantibodies. Benign and malignant thymomas, characterised by major alterations in T cell tolerance related to cortical thymic hyperplasia, are most frequently associated with myasthenia gravis (MG) [204]. There is also an association between thymoma and CNS autoimmunity (most commonly limbic encephalitis), a group of syndromes collectively referred to as thymoma-associated paraneoplastic encephalitis (TAPE) [205–209]. There have also been case reports of thymoma associated with SPSDs [210–212].

Thymectomy has been demonstrated to constitute an effective treatment for MG even in cases not associated with thymoma, consistent with the centrality of thymic dysfunction in MG [213]. Importantly, clinical improvement after thymectomy has also been reported in some cases of TAPE, showing that continuous thymic output of CNS antigen-specific T cells may play a significant role in CNS autoimmunity [205–209]. MG is also associated with susceptibility to NMOSD, particularly following thymectomy [214]. As most patients harbour anti-AQP4 antibodies years before thymectomy and disease onset, this risk appears to be independent of CNS-reactive antibody production and may instead reflect direct precipitation of CNS autoimmunity by the abrupt loss of thymopoiesis, possibly due to loss of thymic tT_reg_ cell output.

Despite major translational insights from preclinical findings, clinical interest in the potential for thymectomy as treatment for MS has been historically lacking after an early trial identified no benefit for thymectomy in patients with relapsing-remitting disease and showed worsening of clinical status in patients with chronic progressive disease [215]. Yet, the small number of patients and invasiveness of thymectomy limit interpretation of these results. More recently, there have been reports of significant clinical improvement after thymectomy in patients with concurrence of MG and MS, although the nature of this improvement is not clear [216]. Due to the lack of large-scale trials of thymectomy in MS patients, evidence of therapeutic effectiveness (or lack thereof) remains observational.

Since clinical improvement after MG or TAPE has been seen after thymectomy in adults, it is likely that modulation of thymoiesis into adulthood offers a potential useful therapeutic avenue.

## Evidence from effects of therapeutic strategies

Just as alterations in thymic function can lead to pathophysiological features of CNS autoimmunity, successful therapeutic amelioration of CNS autoimmune disease can be associated with measurable changes in thymic T cell tolerance.

**Fig. 5:**
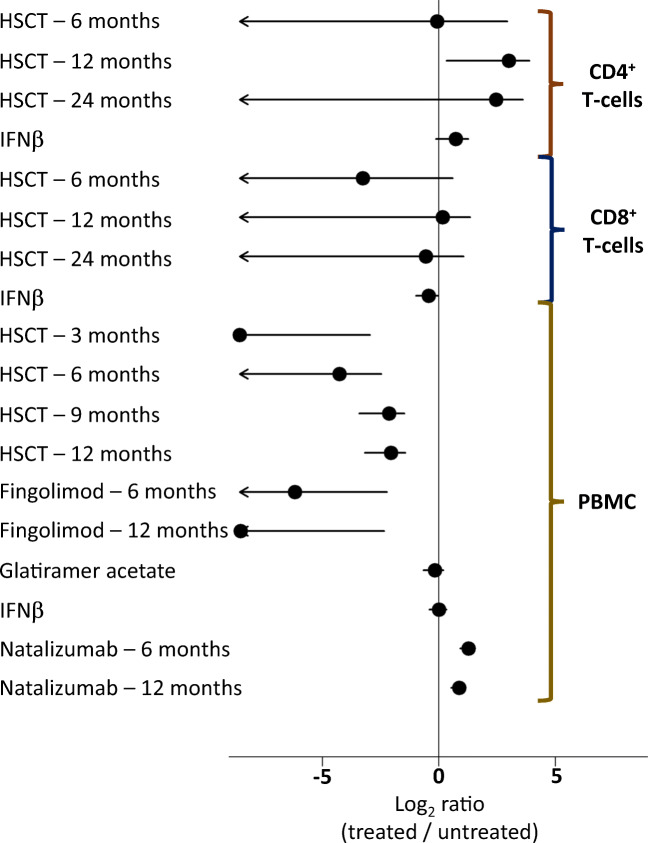
Measuring T cell receptor excision circles in MS treatments. A forest plot of individual studies. IFNβ, interferon-beta; HSCT, haematopoietic stem cell transplant; PBMC, peripheral blood mononuclear cells

Comparative studies on treated versus untreated MS patients have sought to determine effects of different treatment strategies on numbers of TRECs and/or CD31^+^ RTE cells in peripheral blood mononuclear cells (PBMC) and in the CD4^+^ or CD8^+^ T cell compartments specifically (Fig. 5) [154, 156, 159–161, 217, 218]. The results of these have shown few consistent findings, although T cell subtype-specific effects would not be detected in these analyses.

Studies in MS patients subject to autologous haematopoietic stem cell transplantation (HSCT) show that an initial reduction in TREC levels is followed by recovery of the peripheral T cell pool over a 2-year course. This reconstituted T cell compartment is characterised by increased frequency of TREC^hi^ CD31^+^ naïve RTE T cells and a broader TCR repertoire [217]. Myelin-reactive T cells that eventually re-emerge in the recovered T cell pool show significantly diminished T_H_17 responses, and this is associated with abrogation of focal inflammatory disease activity and MS relapses [218, 219]. Therefore, modulation of thymopoiesis leading to the appearance of RTE T cells can occur in adulthood and lead to clinically important changes in pathophysiological features of CNS autoimmunity.

Large and sustained increases in TREC levels are also observed during monoclonal antibody treatment with natalizumab, pointing to a therapeutic association with altered thymopoiesis [160]. In contrast, IFNβ and glatiramer acetate therapy have little effect on TREC levels [154, 156, 159] and major decreases in thymic T cell output are observed during immunomodulatory fingolimod therapy [161].

Evidence from treatment effects thus also points to potential therapeutic benefit of modulating thymopoiesis in CNS autoimmune diseases. More detailed functional phenotyping of RTE T cells associated with altered CNS inflammation is necessary in order to understand how changes in thymopoiesis are linked to reduction in the mechanisms of CNS autoimmunity.

## Therapeutic implications and future directions

The modulation of thymopoiesis has unique potential as a source of novel therapies for CNS autoimmune diseases [13]. While thymectomy constitutes the most direct approach, its routine application is unlikely since gross thymic abnormalities (e.g. thymoma, thymic hyperplasia) are far less frequent in CNS autoimmune diseases than in conditions for which thymectomy is an established treatment, such as MG [204, 213].

### Modulation of intrinsic and environmental factors for thymopoiesis

A less invasive approach to reduce the risk of CNS autoimmunity and improve the effects of disease-modifying therapies could be to artificially manipulate intrinsic and environmental factors for thymic selection, in order to increase the elimination of autoreactive, potentially encephalitogenic T cells and promote generation of CNS-specific tT_reg_ cells.

If intrinsic defects in thymic selection play an important role in CNS autoimmunity, complete renewal of the developing thymocyte pool may yield therapeutic benefit. Clinical studies have indeed shown that, in relapsing-remitting MS patients, nonmyeloablative chemotherapy with anti-thymocyte globulin followed by autologous HSCT is associated with post-transplant improvement in disability scores, neurological function and CNS lesion volume, as well as prolonged time to disease progression compared to immunomodulatory therapy alone [220–222]. While these findings support modulation of thymopoiesis as a viable treatment for CNS autoimmunity, their interpretation is limited by evidence that immune system reconstitution by anti-thymocyte globulin does not just reset thymocyte selection but depletes the peripheral T cell pool as well. The role of tT_reg_ output in post-HSCT improvement is also unclear, although HSCT is broadly associated with at least a transient increase in T_reg_ numbers [223]. While CNS-specific T_reg_ cells reduce neuroinflammation in animal models, development of protocols for efficient generation of human CNS-specific T_reg_ cells and trials in MS patients to determine the influence of these cells on CNS inflammatory profiles are required in order to effectively assess the therapeutic viability of T_reg_ cell–based approaches for human CNS autoimmune disease.

With increased understanding of how thymic tolerance changes in health and disease, it may also be possible to develop preventative strategies that target environmental factors that modulate thymopoiesis in early childhood to reduce risk of CNS autoimmunity in later life. Nutritional factors may play a role: both zinc and vitamin D supplementation has been shown to modulate thymopoiesis in mice [224–226]. However, evidence that vitamin D supplementation improves the course of MS is lacking [227]. Given the association of obesity and premature thymic involution, nutritional strategies aimed at control of BMI and adiposity may also have a protective effect for CNS autoimmunity [228].

In general, growing evidence that metabolism-, sex hormone– and ageing-related factors can have pervasive influences on thymic output and thus the composition of peripheral T cell pools suggests that multiple molecular pathways modulate thymopoiesis and could be preventatively targeted in patients, such as those with strong family history, at high risk for CNS autoimmunity [229, 230]. Large-scale studies, in which quantitative effects of manipulating specific environmental factors on thymopoiesis are measured, would need to be conducted before the therapeutic potential of these factors could be effectively assessed.

### Induced pluripotent stem cell–derived artificial thymic organoids

An alternative and technologically more complex approach to modulate thymopoiesis is the in vitro differentiation from host-derived human induced pluripotent stem cells (iPSCs) of thymic epithelial progenitors (TEPs), which mature into functional TECs upon transplantation into the recipient. More specifically, artificially bioengineered thymic epithelial tissue can be combined with biocompatible 3D scaffolds that mimic the organisation of thymic extracellular matrix to support ex vivo or even in situ generation of artificial thymic organoids (ATOs) [231–234].

Different studies have shown that ATOs can be generated from animal- and human-derived iPSCs or embryonic stem cells (as well as mature postnatal TECs) and can potentially support thymopoiesis in vitro [235–238]. Moreover, findings in athymic nude mice suggest ATO transplantation can effectively promote central T cell tolerance (i.e. reduce allograft rejection) [239].

Importantly, this approach can be combined with genetic manipulation of grafted autologous TEPs to ensure that their TEC progeny expresses desired or putative autoantigens and can thus limit thymic escape of potentially pathogenic T cells by fostering their clonal deletion or differentiation into antigen-specific tolerogenic tT_reg_ cells. The potential efficacy of this strategy for CNS autoimmune diseases is supported by a proof-of-concept study in a preclinical model, in which transplantation of embryonic stem cell–derived TEPs engineered to express MOG rendered mice resistant to later EAE induction through deletion of MOG-autoreactive T cells and generation of MOG-specific T_reg_ cells [240].

Nonetheless, there are key limitations in current understanding of human thymic function that limit future therapeutic applicability of iPSC- and ATO-based approaches for CNS autoimmunity. Firstly, available in vitro models of human thymopoiesis and TCR repertoire selection are incomplete. Development of more sophisticated stem cell–derived thymic models, which can reliably recapitulate complexities of TEC function (especially transcriptional control of TRA gene expression) as well as the roles of intrathymic DCs and B cells, is required before clinical investigations can be pursued.

Furthermore, the less prominent role of thymopoiesis in adult maintenance of the peripheral T cell pool in humans than rodents is a potential obstacle to clinical translation of ATO-based strategies [146]. However, the detection of CNS-specific RTE T cells following reconstitution of the peripheral T cell compartment by autologous or allogeneic HSCT in MS patients demonstrates that substantial potential for therapeutic targeting of thymopoiesis can be present in adulthood [218].

Finally, the correlation between intrathymic levels of CNS autoantigen transcripts, synthesis of CNS peptide antigens in TECs and actual presentation to thymocytes of potentially encephalitogenic TCR epitopes is itself only partly understood. Detailed characterisation of the TEC peptidome through recently developed high-throughput proteome screening assays is required to complement data from transcriptomic studies and thus resolve the proportion of CNS antigens that are effectively presented to developing thymocytes [241]. In turn, this would allow to evaluate more accurately the potential clinical benefits for CNS autoimmunity of approaches, such as ATO-based strategies, aimed at therapeutic modulation of the molecular mechanisms of thymic T cell tolerance.

### TCR clonality of RTE T cells as a measure of treatment effect and relapse risk

An increased consideration of the role of the thymus in CNS autoimmunity in clinical settings could provide an important complement to existing therapies for CNS autoimmunity. In particular, advances in transcriptomic techniques could make the analysis of TCR clonality of recent thymic emigrant T cells a valuable tool to monitor the efficacy of available treatment approaches.

Population-level transcriptomic analysis of peripheral T cell pools allows to identify overrepresentation of specific TCRβ chain locus rearrangements, which gives an indication of overall clonal diversity in peripheral T cells and allows to detect disease-relevant, clonally expanded T cell populations (e.g. in MS). Yet, these approaches cannot determine the specific TCR α- and β-chain pairings in individual cells, which is required to understand TCR antigen specificity and clonality. By contrast, single-cell sequencing approaches make it possible to reconstruct full-length, α-β paired TCR sequences from the RNA sequencing data of individual T cells [242]. Such precise characterisation of the TCR repertoire allows the identification of clonal relationship between T cells and, most importantly, to predict their functional phenotype (e.g. effector/memory) and TCR ligand specificity.

Two main clinical applications can be envisaged for this approach. Firstly, comparing findings from TCR repertoire sequencing of RTE T cells in patients with CNS autoimmune diseases before and after treatment allows to detect whether CNS-reactive, pathogenic T cell clones persist or have been successfully eliminated. In turn, the detection of residual or re-emerging pathogenic T cell clones gives an indication of treatment failure, allows to faithfully predict the occurrence of relapses and may direct the choice of alternative therapeutic approaches. Secondly, single-cell analysis of TCR clonality may allow the identification of novel antigens (e.g. due to epitope spreading) involved in the initiation or continuance of CNS autoimmune responses [243].

As for other strategies, a major knowledge gap limits the potential for testing the practicality of single-cell sequencing-based treatment approaches in preclinical disease models and patient-based studies. Specifically, future research should focus on providing a more detailed description of how thymic antigen presentation, thymopoiesis and the TCR repertoire of RTE T cell populations vary as a function of age (in particular with thymic senescence), as well as on examining the impact on CNS inflammation of the different functional phenotypes of CNS-specific RTE T cells that emerge after therapeutic haematopoietic stem cell transplantation for CNS autoimmune diseases.

## Conclusions

Research into the roles of central tolerance in human CNS autoimmunity has considerably lagged behind research into peripheral tolerogenic mechanisms. Nonetheless, evidence from both preclinical models and studies in human patients over the last two decades has suggested a role for the thymus in susceptibility to and severity of CNS inflammation, and therefore in the risk, pathogenesis, progression and response to treatment of CNS autoimmune disease, in particular MS.

There are still major gaps in our understanding and ability to measure how thymopoiesis and central T cell tolerance change during health and disease, as well as in our ability to discriminate the influences of central tolerance induction and peripheral tolerogenic processes in pathophysiological features of CNS inflammation. Critically, a detailed and comprehensive functional phenotyping of all innate and adaptive immune cells isolated from the CNS is still lacking, although is beginning to emerge from recent studies [244]. Understanding of this would be fundamental to a clearer elucidation of the relationship between the CNS and the peripheral T cell compartment, which may resolve why the CNS is resistant to autoimmunity even in the presence of disrupted thymic selection (e.g. in APS-1). In turn, these knowledge gaps significantly limit the potential for translating the modulation of thymic selection into viable therapeutic strategies for CNS autoimmune disease. The promise of these therapeutic approaches should act to stimulate further research in this area.

In the next decade, studies providing an improved understanding of the roles of thymic tolerance in autoimmune diseases of the CNS may support the emergence of novel interventions with greater efficacy and a lower risk of adverse effects than currently available therapeutic options.

## Data Availability

Not applicable
